# Recurrent Painful Perianal Subcutaneous Angiomatoid Fibrous Histiocytoma: A Case Report and Review of the Literature

**DOI:** 10.1097/MD.0000000000000202

**Published:** 2014-12-02

**Authors:** Xiangyi Kong, Dachun Zhao, Guole Lin, Jiaolin Zhou, Quancai Cui

**Affiliations:** From the Department of Neurosurgery (XK); Department of Pathology (DZ, QC); and Department of General Surgery (GL, JZ), Peking Union Medical College Hospital, Chinese Academy of Medical Sciences, Dongcheng District, Beijing, P.R. China.

## Abstract

Angiomatoid fibrous histiocytoma (AFH) is a rare, low-grade malignant soft-tissue tumor most commonly occurring in the extremities of children and young adults and has a low potential of local recurrence and metastasis. Here, we present a case of recurrent subcutaneous perianal AFH. After an initial diagnosis as a sebaceous cyst, we were able to use immunohistochemical findings to correctly identify the mass as an AFH. The patient was effectively treated after 3 surgical resections. This case emphasizes the need to correctly diagnose soft-tissue tumors using a variety of diagnostic modalities to ensure that the patient receives proper treatment.

## INTRODUCTION

Angiomatoid fibrous histiocytoma (AFH) is a rarely metastasizing soft-tissue tumor of low-grade malignant potential that mostly affects children and young adults.^1^ The clinical presentation of affected patients is often similar to other diseases, such as perianal sebaceous cyst and lipomyoma, and the pathological review is not always clear. Because of the potential for misdiagnosis, molecular diagnostics are increasingly being used to detect gene fusions characteristic of AFH.^2^ About two-thirds of cases occur at sites with abundant lymph nodes, such as the antecubital fossa, popliteal fossa, axilla, and inguinal and supraclavicular areas.^3^ AFH is often associated with systemic symptoms such as anemia, fever, weight loss, enhanced cytokine production, polyclonal gammopathy, and a Castleman disease-like lymphadenopathy.^4^ Local recurrence is more likely in deeply situated tumors and those with infiltrative margins.^5^ Surgical resection is the most common management and can effectively control local recurrences and metastases. Only a few cases have been reported demonstrating AFH arising in sites other than the deep dermis and subcutaneous tissue of the extremities, trunk, head, and neck.^6^ Xiang et al^7^ reported a case of AFH arising from the retroperitoneum.

Herein, we present a case of a 35-year-old woman suffering from AFH presenting as a recurrent perianal subcutaneous mass, who underwent 4 surgeries. To the best of our knowledge, this is the first reported case of AFH presenting in this manner. Because the rarity of AFH often leads to the misdiagnosis of either a reactive lesion or a benign or malignant tumor,^8^ we also review the latest literature on the etiology, diagnosis, and treatment of AFH.

## CASE REPORT

**FIGURE 1:**
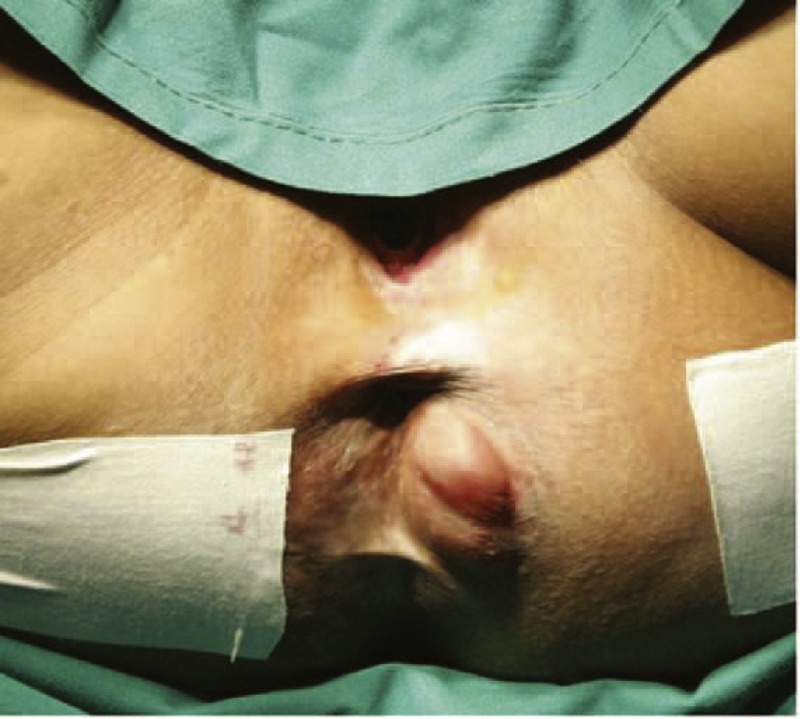
The patient's recurrent perianal subcutaneous AFH mass. Although the mass was only approximately 2 × 1 cm during the initial visit, it grew to approximately 4 × 2.5 cm 1 month later. AFH = angiomatoid fibrous histiocytoma.

A 35-year-old woman was admitted to our hospital complaining of a perianal subcutaneous mass. Two years ago, we treated her for a similar mass that we surgically resected (unfortunately, the tissue was not taken to do pathological examination). Both masses were similar in tenderness, texture, and mobility. The only difference of the current mass was in its location, on the left side of her anus and exposed to air. During this visit, the mass was the size of a peanut. Approximately 1 month later, however, it grew to approximately 4 × 2.5 cm (Figure 1). She frequently felt pain whenever sitting. The patient did not exhibit fatigue, fever, chills, or pruritus and was not taking any medications. She noticed a slight weight loss despite a normal appetite. The patient's only previous surgery was a Cesarean section in 2006. Her husband and 1 son are healthy. We did not identify any special circumstances regarding her family history, personal history, and menstrual history related to her presentation. Upon physical examination, she was afebrile with blood pressure of 126/77 mm Hg and a regular pulse of 89 bpm. Her abdomen was soft and without tenderness. Bowel sounds were normal at 3 to 5/min. A well-circumscribed, tender, soft, and ovoid mass measuring 3 × 4 cm in the subcutaneous adipose tissue was located about 2 cm from outside the anal edge at the 9-o’clock position of the knee–chest position. The digital rectal examination did not reveal any remarkable characteristics. Blood, urine, and stool tests also did not reveal anything abnormal. A test for liver and kidney function did not show any abnormal changes. Some serum cancer-associated biomarkers were tested, among which CA724 was much higher at 30.1 U/mL (normal level < 6 U/mL).

**FIGURE 2:**
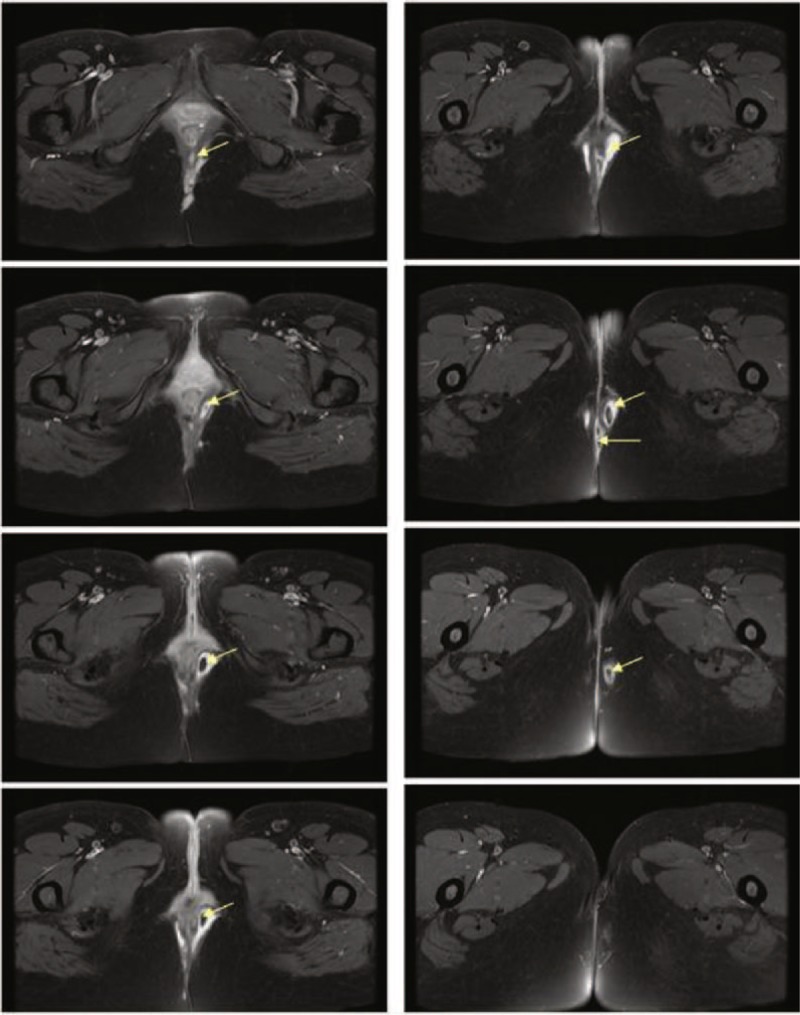
MRI of the patient's pelvic cavity revealed small multifocal areas (arrows) on the left, right, and back side of the anus. These foci had a weaker intensity on the T1WIs and were more heterogeneous with stronger intensity on the T2-weighted images. Note that the left side of the pelvic cavity (L) appears larger than the right side (R). MRI = magnetic resonance imaging, T1WI = T1-weighted image.

A computed tomography (CT) angiography showed no abnormalities of the abdominal arteries and revealed an abnormal soft tissue mass surrounding the anus. Magnetic resonance imaging (MRI) of her pelvic cavity revealed small multifocal areas on the left, right, and back side of the anus (Figure 2). These signals were less intense on the T1-weighted image (T1WI) and slightly heterogeneous and more intense on the T2-weighted image (T2WI) with variegated internal and nodular peripheral gadolinium enhancement. The left side of the pelvic cavity appeared larger than the right side in these images. Together, these images are representative of the features of a perianal abscess.

**FIGURE 3:**
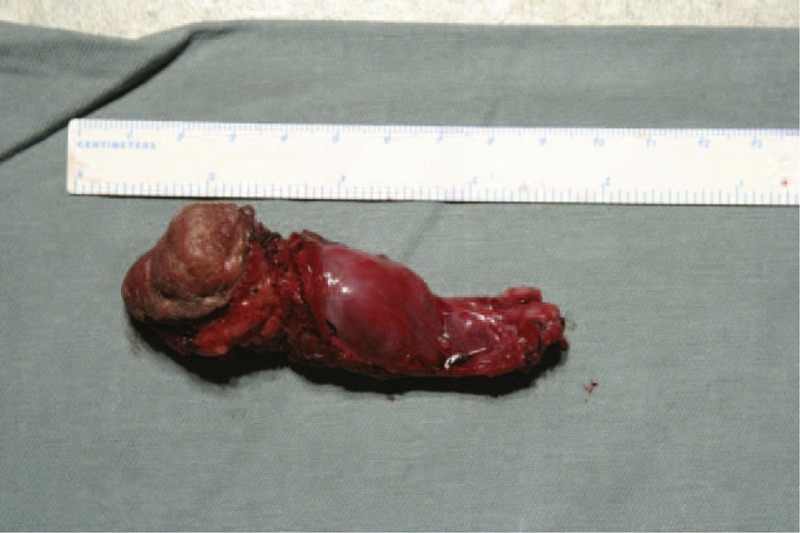
A firm hemorrhagic mass measuring 8.0 × 4.0 × 3.5 cm was removed from the patient.

Given this diagnosis, we performed a retroperitoneal tumor resection and transverse colostomy and removed a firm hemorrhagic mass measuring 8.0 × 4.0 × 3.5 cm (Figure 3). Pathological examination revealed that the mass infiltrated the surrounding striated muscle. Furthermore, this mass showed characteristic features of AFH including irregular aggregates of histiocyte-like cells associated with prominent stromal hemorrhaging of nonendothelial-lined cysts, a dense hyaline fibrous pseudocapsule, peripheral lymphoplasmacytic infiltration with germinal centers, and surrounded by a chronic inflammatory response. These cells were positive for desmin, smooth muscle actin, and a scattered distribution of CD68; negative for anaplastic lymphoma kinase; had a proliferative index of 10% (based on Ki-67 positivity). We suspected this mass was a recurrence of the previously removed one. After the operation, the patient's pain was relieved, and she was able to sit down comfortably.

Four months later, the patient returned to PUMCH for stoma apothesis surgery. After an ultrasound examination, a solid and cystic mass with an unclear border was found again on the left rear of her anus in the vicinity as the last mass, measuring 3.7 × 1.6 × 1.5 cm. Based on her previous history, we suspected it a local recurrence of AFH. After approval from the patient's family, we surgically resected the mass instead of treating the patient for stoma apothesis. The operation was completed without complication. Postoperative pathology confirmed this mass was a local recurrence of AFH. After 3 months, the patient underwent a stoma apothesis surgery after a colon patency test. Staging CT and MRI of the abdomen and pelvis showed no evidence of metastasis and recurrence. To date, the condition of the patient is stable.

## DISCUSSION

According to the World Health Organization (WHO) Classification of Tumours of Soft Tissue and Bone, AFH is described as an intermediate soft-tissue tumor that is often locally aggressive.^9^ Occasionally, AFH gives rise to distant metastases.^1^ The risk of such metastases appears to be <2% and is not reliably predictable based on histopathological findings. AFH was first proposed as a distinct fibrohistiocytic tumor of children and young adults simulating a vascular neoplasm according to 41 examples of an unusual fibrohistiocytic sarcoma described by Enzinger^10^ in 1979 and termed as “angiomatoid malignant fibrous histiocytoma.” The tumor was later formally renamed AFH because of its slow growth and rare metastasis according to WHO classification of soft-tissue tumors concerted in 2002.^11^

Although AFH is most commonly associated with the extremities, there have been a number of reported cases of AFH arising at additional sites such as the mediastinum, bone, intrapulmonal tissue, intracranial tissue, retroperitoneum, lung, vulva, and ovary.^6^ To the best of our knowledge, our case is the first report to describe a recurrent perianal subcutaneous AFH with no usual systemic symptoms such as anemia, fever, and so on.

Despite previous studies, it is still hard to differentiate AFH from malignant fibrous histiocytoma, sarcoma, or other tumors with imaging alone.^12^ On CT images, these confusable heterogeneous soft-tissue mass in the extremities are usually isodense to muscle with high attenuating areas corresponding with hemorrhaging. MRI is superior to CT in depicting fluid–fluid levels within the cystic component of the mass, indicative of intralesional hemorrhage. AFH lesions are often homogeneously hypointense (isointense to muscle) on the T1WI and heterogeneously hyperintense on the T2WI.^1^ In our case, the mass on CT is slightly hypodense to muscle and its MRI fit with the reported findings.

AFHs have a broad range of histological features. Typical AFHs are characterized by 3 microscopic findings: solid arrays or nests of histiocyte-like cells, hemorrhagic cyst-like spaces, and aggregates of chronic inflammatory cells. Multifocal recent and old hemorrhages are a striking feature of this tumor. These spaces resemble vascular spaces, but they are not lined by endothelium. Inflammatory cells present include lymphocytes and plasma cells. A thick pseudocapsule and occasional germinal centers give this tumor a resemblance of a lymph node.^13^

In addition to features that have been previously described such as nuclear pleomorphism, increased mitotic activity, atypical mitotic figures, reticular, myxoid patterns, lymphoplasmacytic infiltration, pseudovascular spaces filled with blood, round cell morphology, and spindle cell patterns.^14^ Bohman et al^4^ reported a series of cases with perineurioma-like areas, with eosinophils in the associated inflammatory infiltrate, and with numerous eosinophils. To clarify the cellular differentiation features of AFH, Hasegawa et al^15^ once examined 4 cases of AFH by clinicopathologic, immunohistochemical, and ultrastructural analyses. Seventy-five percent examined by electron microscopy had a mixture of fibrohistiocytic, myofibroblastic, and undifferentiated cells containing cytoplasmic processes and dense core granules. The authors pointed that AFH has a variety of immunophenotypes, including histiocytic, myofibroblastic, epithelial, and neural and may occasionally have predominantly round cell morphology.^15^

The immunohistochemical features sometimes present a unique immunophenotype. For instance, approximately 50% to 60% of AFH lesions coexpress desmin, EMA, CD68, and CD99 but are generally negative for keratins, S-100 protein, CD34, and follicular dendritic cell markers (CD21 and CD35).^2^ Cytogenetic analysis of our case demonstrated positivity for desmin, CD68, and CD99.

Because the role of immunohistochemistry in establishing the diagnosis of AFH is still limited, additional markers will be important to distinguish this tumor. Recently, the molecular pathogenesis of AFH has become clarified by the discovery of fusion genes resulting from various chromosomal translocations, including the most prevalent EWSR1-CREB1 derived from t(2;22)(q33;q12),^16^ followed by EWSR1-ATF1 from t(12;22)(q13;q12),^17^ and the least common FUS-ATF1 from t(12;16)(q13;p11).^18^ Thway et al expanded the clinicopathological spectrum of AFH by characterizing 13 AFH cases by fluorescence in situ hybridization (FISH) and reverse transcription polymerase chain reaction (RT-PCR). Besides the EWSR1 gene rearrangement detected in 4 cases by FISH, EWSR1-CREB1 fusion was confirmed in 9 cases, including a schwannoma-like AFH, and a EWSR1-ATF1 fusion was detected in a myoepithelioma-like AFH.^16^ These studies suggest that combinatorial FISH and RT-PCR will likely be diagnostically powerful tools to identify AFH by evaluating EWSR1-CREEB1, EWSR1-ATF1, or FUS-ATF1 fusions.^2^

It is important to correctly diagnosis AFH patients to avoid overtreatment when misinterpreted as a more aggressive tumor such as malignant fibrous histiocytoma, nodular Kaposi sarcoma, Ewing sarcoma, and rhabdomyosarcoma. Considering its potential for recurrence and metastasis, it is also important to avoid undertreatment by misdiagnosing it as a sebaceous cyst, perianal abscess, aneurysmal benign fibrous histiocytoma, or inflammatory pseudotumor.^19^ In the currently reported case, the initial diagnosis was as a sebaceous cyst because of the resemblance in appearance. The imaging studies we obtained were not diagnostic, but a pathological review of the immunohistochemistry confirmed our AFH diagnosis.

## CONCLUSION

We report an uncommon case of AFH located around the anus without systematic symptoms. This patient suffered a frequent recurring AFH mass and underwent 3 surgical resections. AFH is a rare disease that is often initially misdiagnosed. It is a challenge to avoid overtreatment or undertreatment. Our case emphasizes the need to correctly diagnose soft-tissue tumors using a variety of diagnostic modalities to ensure that the patient receives proper treatment.
